# Effects of Random Feeding Schedule on Pacing in Asiatic Lions (*Panthera leo persicus*)

**DOI:** 10.1002/zoo.21857

**Published:** 2024-09-30

**Authors:** Leonie Fitskie, Jeroen Kappelhof, Filipe Cunha

**Affiliations:** ^1^ Behavioral Ecology Group, Department of Animal Sciences Wageningen University & Research The Netherlands; ^2^ Royal Rotterdam Zoological & Botanical Gardens Rotterdam The Netherlands

**Keywords:** captive felids, carnivores, pacing, random feeding schedule

## Abstract

For large felids in the wild, intervals between feeding events are irregular since these depend on prey availability and individual hunting success. In contrast, many zoos feed their large felids on fixed schedules. Predictable feeding schedules could induce food anticipatory behavior, like pacing, and randomizing feeding schedules may reduce this anticipatory behavior. Furthermore, random feeding schedules create more variability in the animals environment, which may reduce frustration or boredom. This case study aimed to investigate the effects of a random feeding schedule on pacing behavior of captive Asiatic lions (*Panthera leo persic*a) in the Rotterdam Zoo, The Netherlands. Study animals were observed directly under two treatments: “fixed feeding” (i.e., two fixed feeding days per week) and “random feeding” (i.e., two semi‐randomly picked feeding days per week). Under the random feeding schedule, the mean pacing proportion of lions significantly decreased compared to the fixed feeding schedule. These findings suggest that a random feeding schedule could reduce pacing, either of anticipatory or stereotypic nature, in Asiatic lions. Random feeding schedules do pose a few logistic challenges, such as zookeeper schedules and transport or storage of feed. Nevertheless, random feeding could be a relatively inexpensive strategy to reduce pacing.

## Graphical abstract

In the wild, the timing between feeding events for large felids is irregular; however, in many zoos, those animals are fed in predictable fixed schedules. Given that unpredictability could improve welfare, in this case study, we investigated the effects of a random feeding schedule on pacing behavior of captive Asiatic lions (*Panthera leo persic*a) in the Rotterdam Zoo, The Netherlands. Under the random feeding schedule, the mean pacing proportion of lions significantly decreased compared to the fixed feeding schedule.

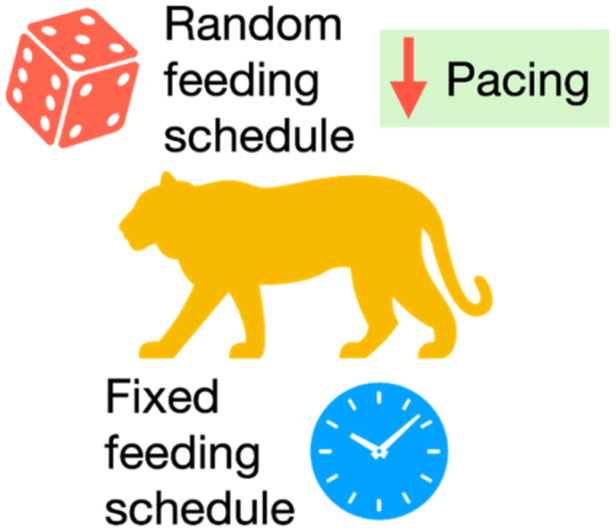

## 1 Introduction

In the wild, feeding intervals of large felids depend on prey availability and individual hunting success (M. Sunquist 2010). Because of the opportunistic hunting style of large felids, these feeding intervals are irregular. For example, feeding intervals ranging from 8.3 up to 10.6 days were found in Leopards (*Panthera pardus*) (Stein, Bourquin, and McNutt 2015). In Tigers (*Panthera tigris*), reported feeding rates fluctuate between one kill in 5 days and one kill in 9 days (Miller et al. 2013; M. E. Sunquist 1981). Feeding intervals are related to environmental characteristics (e.g., prey availability), thus varying between prides (e.g., in African lions [*Panthera leo leo*] [Stander 1992]).

In contrast, zoos' feeding schedules for large felids are often fixed and predictable, i.e., feeding occurs at fixed days or even at fixed times, according to fixed diets (Altman, Gross, and Lowry 2005). Feeding is a rewarding appetitive event and animals in captivity have no control over it. Literature suggests that the predictability over such rewarding events reduces negative effects on welfare (Podturkin, Krebs, and Watters 2024). Under a predictable feeding schedule, brown capuchins exhibited higher levels of social behavior and were overall more active compared to under a random feeding schedule (Ulyan et al. 2006). Furthermore, a random feeding schedule induced weight loss, reduced food intake, and excessive wheel running in rats (Pèrez‐Padillia et al. 2010). Delays in predictable feedings can increase stress and prolonged periods of abnormal behavior (Waitt and Buchanan‐Smith 2001).

In fixed feeding schedules animals can predict feeding events based on internal (e.g., hunger level) or external (e.g., zookeeper presence) cues, and perform food anticipatory behavior around feeding times. Reported food anticipatory behaviors include higher activity/arousal (Bishop 2013), higher levels of pacing (Lyons, Young, and Deag 1997), and elevated levels of inactivity (Bloomsmith and Lambeth 1995). Anticipatory behavior can reflect that an animal perceives its environment as predictable (Podturkin, Krebs, and Watters 2024). It also shows sensibility to rewards and may indicate a positive affective state (Watters 2014). However, in a study with Polar Bears individuals performing anticipatory behavior also showed higher levels of pacing (Cless and Lukas 2017).

While some studies describe a positive effect of predictability and fixed feeding schedules, others point out environmental predictability as possible cause of unvarying, abnormal behavior (e.g., Lewis et al. 2006). For example, a fixed feeding schedule was found to be a predictor for stereotypic behavior in a study with Cheetahs from multiple zoos (Quirke, O'Riordan, and Zuur 2012). Random feeding schedules, in which feeding events are randomly determined, make the environment of captive animals more unpredictable and varied. This could potentially decrease anticipatory behavior and stereotypic pacing in general. Current evidence suggests that pacing decreased when switched to a random feeding schedule in Tigers (Jenny and Schmid 2002), Leopard Cats (Shepherdson et al. 1993), and Cheetahs (Quirke and O'Riordan 2011). Moreover, shifting from fixed to random feeding schedules is reported to induce more natural behavior. Compared to a fixed schedule, random feeding increased natural exploratory behavior in Red Foxes (Kistler et al. 2009) and Leopard Cats (Shepherdson et al. 1993), and increased territorial marking behavior in Coyotes, reaching similar levels of marking frequencies of wild Coyotes (Gilbert‐Norton, Leaver, and Shivik 2009). Thus, random feeding schedules could be a tool to reduce pacing and stimulate natural behaviors in large felids.

Implementing random feeding schedules could pose logistical challenges for zoos. These challenges may include complicated zookeeper schedules or challenges in planning feed transportation or ‐storage. Nevertheless, random feeding schedules could be implemented with low or no additional costs and could therefore be a relatively inexpensive enrichment strategy.

This research aimed to investigate the effects of a random feeding schedule on pacing behavior of large felids in a case study with captive Asiatic lions (*Panthera leo persicus)*. We observed the study animals under a fixed feeding schedule (two fixed feeding days per week), followed by observations under a random feeding schedule (two feeding days picked semi‐randomly per week). Pacing was expected to decrease under the random feeding schedule compared to the fixed feeding schedule.

## 2 Methods

We conducted the study in a group of four captive‐born female Asiatic lions (*Panthera leo persicus*) housed in Diergaarde Blijdorp (Rotterdam Zoo), the Netherlands. The enclosure has a naturalistic design with free access to an on‐display outdoor area and an on‐display indoor area. The lion group included a female (Lalana, age 11) and her three daughters (Asha, Mette, Reena, all age 3). Rotterdam Zoo still kept two older lionesses, however, they were not included in this study. They were kept in a separate, off‐display, adjacent enclosure. The lionesses in the two different enclosures could hear and smell each other but did not have visual contact.

We exposed the animals to two treatments: a fixed feeding schedule (baseline) and a random feeding schedule (treatment). Each treatment lasted 6 weeks, starting with a 3‐week acclimatization period, followed by a 3‐week observation period. During the acclimatization period, no observations occurred, so the animals could get accustomed to the next feeding schedule. During both treatments, the zoo allowed no visitors due to COVID‐19 restrictions. On May 19, 2021, the restrictions were lifted, thus visitors were present during the last three observation days of the random feeding treatment.

During the fixed feeding schedule, the animals were fed on two fixed feeding days per week. On Thursdays the lions received 10 kg of meat per lion (e.g., boneless meat, goose, rabbit, or tripe) and on Sundays the lions received 10 kg of bone‐containing meat per lion. During feeding, the lions were kept into the indoor enclosure and zookeepers distributed the meat over the outdoor enclosure. Feeding times occurred between 12:00 h and 16:00 h, depending on the zookeepers' schedules. On the observed feeding days in this treatment, lions were able to access their feed at 13:40, 15:55, 13:44, and 15:48 h.

During random feeding, we semi‐randomly picked two feeding days per week (the minimum interval between feeding days was 2 days due to animal satiety). Feeding occurred randomly between 9:30 h and 16:30 h, depending on the schedule of the zookeepers. Lion diet composition was the same as during the fixed feeding schedule. On the observed feeding days in this treatment, lions were able to access their feed at 14:00, 9:55, 14:07, and 9:40 h.

After both treatments, we observed the lions under a fixed feeding schedule again, following an ABA‐design. During this period, animals received food on the same fixed days as during the first fixed feeding schedule, according to the same diet composition. The zoo adjusted the lion management during this period: Feeding occurred before 9:30 h and during feeding, the zookeepers kept the on‐display lion group (Lalana and offspring) in the outdoor area. During this phase the two off‐display older lionesses had access to the on‐display indoor enclosure. The two lion groups could hear and smell each other, but visual contact was limited to the small opening underneath the enclosure doors. Between 13:00 h and 14:00 h, the zookeepers locked the older lionesses into the off‐display enclosure again and the on‐display lion group regained access to the on‐display indoor enclosure. In addition, the COVID‐19 restrictions were lifted during this period, allowing visitors into the zoo. Considering the confounding influence of these changes in feeding times, housing situation, and visitor presence, we decided not to use the data from this period for our study.

Observations took place from March until July 2021. Per treatment, we observed the lions on four feeding days and ten fasting days. We observed the animals from 9:30 h until 12:30 h and again from 13:30 h until 16:30 h. This resulted in a total of 168 h of observations. We used instantaneous scan sampling with 1‐min intervals to record the Lions' behavioral state every 30 min. LF performed all observations. We based the ethogram (Supporting Information S1: Table S1) on Stanton's standardized Felidae ethogram (Stanton, Sullivan, and Fazio 2015) and personal observations during a pilot study.

We grouped the counted state behaviors per category and transformed them into daily proportions (the fraction of scan samples that animal was observed in a specific state over all scan samples per day). We calculated activity budgets with mean daily proportions of state behavior categories. For all statistical analyses we used R statistical software, version 4.3.2 (R core team, 2023).

To analyze pacing behavior we used the proportion of scan samples spent pacing divided by all scan samples per day. To test if pacing was influenced by the feeding schedules, we used a generalized linear model (“glm” function) with a log link function, using a quasi‐Poisson family. We also included the individuals and feeding day (yes or no) as covariates. The fixed feeding schedule was the reference treatment. We used the R package “contrast” (O'Callaghan et al. 2020) to perform contrast analyses and test for differences in pacing between individual lionesses. The contrast analysis shows the difference in means between covariate groups, i.e. the individual lionesses.

## 3 Results

**Figure 1:**
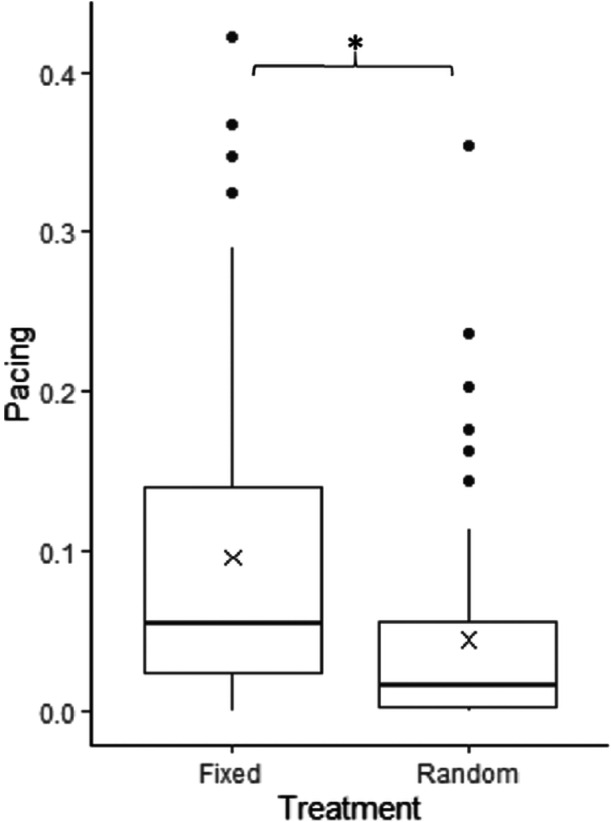
Boxplot of the proportion of scans lions spent pacing overall all scan samples during the fixed and random feeding schedule. Crosses indicate means. *p < .05.

During both treatments, lions were inactive on most scan samples. Mean (±SD) proportions of scans spent on inactive behavior over all scan samples ranged from 0.73 (±0.15) during the fixed feeding schedule to 0.77 (±0.13) during the random feeding schedule (for full activity budgets, see Supporting Information S1: Figures S1 and S2). Between the lionesses there was some variation in proportion of the scans spent pacing over all scan samples. One of the younger lionesses, Asha, displayed the highest pacing proportions over both feeding schedules (0.092 ± 0.11) and the oldest lioness, Lalana, displayed the least pacing (0.041 ± 0.06). Levels of activity and feeding behavior were similar between the individual lionesses for each treatment. Pacing was significantly more present on fasting days (0.089 ± 0.10) than on feeding days (0.033 ± 0.03) (Table 1). Furthermore, the proportion of scans spent pacing over all scan samples significantly decreased during the random feeding schedule (0.045 ± 0.07) compared to the fixed feeding schedule (0.096 ± 0.10) (Table 1, Figure 1).

**Table 1:** Results of generalized linear model for mean proportion of scans lions spent pacing over all scan samples as a function of treatment, individual, and feeding day.

	Estimate	SE	*t* value	Pr(> |*t*|)
Intercept	−1.88	0.20	−9.56	< 0.001*
Fixed versus Random	−0.77	0.22	−3.52	0.001*
Asha versus Lalana	−0.81	0.32	−2.51	0.014*
Asha versus Mette	−0.12	0.26	−0.48	0.634
Asha versus Reena	−0.31	0.28	−1.13	0.259
Fasting versus feeding day	−0.95	0.30	−3.14	0.002*

* Significant *p*‐values (*α* = 0.05).

## 4 Discussion

Overall, our findings suggest that pacing significantly decreased under a random feeding schedule compared to a fixed feeding schedule in four Asiatic lions. Although, on fasting days, lions paced significantly more compared to feeding days.

Similar to these findings, higher pacing proportions on fasting days were also reported in African lions, both in prides on a high‐frequency diet (four to 5 days per week) and on a low‐frequency diet (1 day per week) (Höttges et al. 2019). For several large felid species that were fed every 3 days, pacing levels were higher on fasting days compared to feeding days as well (Lyons, Young, and Deag 1997). These findings suggest that captive carnivores can anticipate on the fact that they will not receive food on fixed fasting days, although they feel appetitive motivation. This may lead to pacing due to frustrating appetitive motivation (Bishop 2013). Contradictory, another group of African lions paced more on feeding days compared to fasting days on a schedule with three random feeding days per week (Altman, Gross, and Lowry 2005). The authors argue that this increase is due to increased anticipatory behavior caused by external cues (the presence of zookeepers or the smell of fresh meat) on feeding days. However, lions in our study were similarly cued by these events on feeding days. Furthermore, feeding occurred in the morning in the study of Lyons, Young and Deag (1997) and on some days in our study, whereas lions only received food in the afternoons in the study of Altman, Gross and, Lowry (2005). Feeding in the morning satiates the animals, which can reduce frustrating appetitive behavior later in the day.

Pacing significantly decreased under a random feeding schedule combined with spatial unpredictability in Leopard Cats and Tigers (Shepherdson et al. 1993; Jenny & Schmid). Additionally, Cheetahs showed a decreasing trend in pacing when daily feeding times were randomized (Quirke and O'Riordan 2011). These findings suggest that a more unpredictable feeding schedule could reduce anticipatory pacing behavior toward food. The overall low proportions that lions spent pacing (less than 0.1) could indicate that pacing is mostly anticipatory rather than stereotypical. Anticipatory behavior can indicate whether an animal perceives an event as predictable or not (Podturkin, Krebs, and Watters 2024) and it may reflect an animal's sensitivity to rewards and a positive affective state (Watters 2014). Therefore, random feeding schedules may decrease anticipatory behavior without tackling the underlying mechanism of frustrative motivation to forage. On the other hand, Polar Bears performing anticipatory pacing showed more intense and more pacing in general compared to Polar Bears not performing anticipatory behavior (Cless and Lukas 2017). The authors suggest that anticipatory pacing could develop into more generalized pacing when an animal is stressed, frustrated, or bored and that tolerating anticipatory pacing could have potential dangers.

Alternatively, the decrease in pacing during random feeding could simply be due to increased variation in the environment. In the wild, large carnivores have to deal with rapidly changing environments, which challenges animals to develop behavioral plasticity (M. Sunquist and Sunquist 2017). In zoos, the environment may be unvarying and predictable, which does not stimulate variable behavior and could lead to unvarying and repetitive behavior like pacing (Lewis et al. 2006; Schneider, Nogge, and Kolter 2014). A random feeding schedule creates more variability, which could reduce boredom and stimulate more varying and functional behavior instead of pacing (Clubb and Mason 2007; Lewis et al. 2006).

On four occasions, two times during the fixed and two times during the random feeding schedule, feeding occurred within 1 h after the observer's afternoon break. This may have caused bias in our data as anticipatory behavior usually occurs in the time shortly before feeding, therefore some anticipatory behavior might have been missed. Furthermore, some bias in our data could have been caused by the presence of visitors on the last three observation days of the random feeding schedule. The absence of visitors during the experiment was atypical and, although currently understudied, visitors may influence enclosure use and pacing behavior of captive carnivores (Suárez, Recuerda, and Arias‐de‐Reyna 2017).

Thus, our findings suggest that random feeding schedules could be a useful tool to decrease pacing in captive carnivores. Shifting from fixed to random feeding schedules may cause logistical problems for feeding management in zoos, e.g., complicated zookeeper schedules or challenges for storage and transportation of feed. However, random feeding does not require (much) additional costs and could therefore be an inexpensive strategy to reduce pacing. Further research should focus on the effects of random feeding schedules on different species of large carnivores, keeping in mind their natural feeding ecology.

## Ethics Statement

The authors have nothing to report.

## Conflicts of Interest

For our conflict of interest, we state that Jeroen Kappelhof works for Rotterdam zoo, where the research was conducted.

## Supporting information

Supplementary information.

## Data Availability

The data sets generated for this study are available on request to the corresponding author.

## References

[zoo21857-bib-0002] Altman, J. D. , K. L. Gross , and S. R. Lowry . 2005. “Nutritional and Behavioral Effects of Gorge and Fast Feeding in Captive Lions.” Journal of Applied Animal Welfare Science 8, no. 1: 47–57. 16004544 10.1207/s15327604jaws0801_4

[zoo21857-bib-0003] Bishop, J. K. 2013. “Predictable Feeding in Zoos: Research Methods and Behavioral Effects.” Doctoral thesis, Plymouth University. https://pearl.plymouth.ac.uk/bitstream/handle/10026.1/1580/2013bishop10019534phd.pdf?isAllowed=y&sequence=1.

[zoo21857-bib-0004] Bloomsmith, M. A. , and S. P. Lambeth . 1995. “Effects of Predictable Versus Unpredictable Feeding Schedules on Chimpanzee Behavior.” Applied Animal Behaviour Science 44, no. 1: 65–74.

[zoo21857-bib-0005] Cless, I. T. , and K. E. Lukas . 2017. “Variables Affecting the Manifestation of and Intensity of Pacing Behavior: A Preliminary Case Study in Zoo‐Housed Polar Bears.” Zoo biology 36, no. 5: 307–315. 28901667 10.1002/zoo.21379

[zoo21857-bib-0006] Clubb, R. , and G. J. Mason . 2007. “Natural Behavioural Biology as a Risk Factor in Carnivore Welfare: How Analysing Species Differences Could Help Zoos Improve Enclosures.” Applied Animal Behaviour Science 102, no. 3–4: 303–328.

[zoo21857-bib-0007] Gilbert‐Norton, L. B. , L. A. Leaver , and J. A. Shivik . 2009. “The Effect of Randomly Altering the Time and Location of Feeding on the Behaviour of Captive Coyotes (*Canis latrans*).” Applied Animal Behaviour Science 120, no. 3–4: 179–185.

[zoo21857-bib-0008] Höttges, N. , M. Hjelm , T. Hård , and M. Laska . 2019. “How Does Feeding Regime Affect Behavior and Activity in Captive African Lions (*Panthera leo*)?” Journal of Zoo and Aquarium Research 7, no. 3: 117–125.

[zoo21857-bib-0009] Jenny, S. , and H. Schmid . 2002. “Effect of Feeding Boxes on the Behavior of Stereotyping Amur Tigers (*Panthera tigris* altaica) in the Zurich Zoo, Zurich, Switzerland.” Zoo Biology 21, no. 6: 573–584.

[zoo21857-bib-0010] Kistler, C. , D. Hegglin , H. Würbel , and B. König . 2009. “Feeding Enrichment in an Opportunistic Carnivore: The Red Fox.” Applied Animal Behaviour Science 116, no. 2–4: 260–265.

[zoo21857-bib-0011] Lewis, M. H. , M. F. Presti , J. B. Lewis , and C. A. Turner . 2006. “The Neurobiology of Stereotypy 1: Environmental Complexity.” In Stereotypic Animal Behaviour. Fundamentals and Applications to Welfare, 2nd edition, edited by G. Mason and J. Rushen , 190–226. Wallingford: CAB International.

[zoo21857-bib-0012] Lyons, J. , R. J. Young , and J. M. Deag . 1997. “The Effects of Physical Characteristics of the Environment and Feeding Regime on the Behavior of Captive Felids.” Zoo Biology 16, no. 1: 71–83.

[zoo21857-bib-0014] Miller, C. S. , M. Hebblewhite , Y. K. Petrunenko , et al. 2013. “Estimating Amur Tiger (*Panthera tigris* altaica) Kill Rates and Potential Consumption Rates Using Global Positioning System Collars.” Journal of Mammalogy 94, no. 4: 845–855.

[zoo21857-bib-0015] O'Callaghan, A. , M. Kuhn , S. Weston , et al. 2020. *Contrast: A Collection of Contrast Methods*. Package version 0.22. https://CRAN.R-project.org/package=contrast.

[zoo21857-bib-0016] Pérez‐Padilla, Á. , P. Magalhães , and R. Pellón . 2010. “The Effects of Food Presentation at Regular or Irregular Times on the Development of Activity‐Based Anorexia in Rats.” Behavioural Processes 84, no. 1: 541–545. 20176093 10.1016/j.beproc.2010.02.007

[zoo21857-bib-0017] Podturkin, A. A. , B. L. Krebs , and J. V. Watters . 2024. “Quantifying Animals' Perception of Environmental Predictability Using Anticipatory Behavior.” Zoo Biology 43, no. 2: 125–135. 38082553 10.1002/zoo.21811

[zoo21857-bib-0018] Quirke, T. , and R. M. O'Riordan . 2011. “The Effect of a Randomised Enrichment Treatment Schedule on the Behaviour of Cheetahs (*Acinonyx jubatus*).” Applied Animal Behaviour Science 135, no. 1–2: 103–109.

[zoo21857-bib-0019] Quirke, T. , R. M. O'Riordan , and A. Zuur . 2012. “Factors Influencing the Prevalence of Stereotypical Behaviour in Captive Cheetahs (*Acinonyx jubatus*).” Applied Animal Behaviour Science 142, no. 3–4: 189–197.

[zoo21857-bib-0020] R Core Team . 2023. R: A Language and Environment for Statistical Computing. Vienna, Austria: R Foundation for Statistical Computing. https://www.R-project.org/.

[zoo21857-bib-0021] Schneider, M. , G. Nogge , and L. Kolter . 2014. “Implementing Unpredictability in Feeding Enrichment for Malayan Sun Bears (*Helarctos malayanus*).” Zoo Biology 33, no. 1: 54–62. 24402968 10.1002/zoo.21112

[zoo21857-bib-0022] Shepherdson, D. J. , K. Carlstead , J. D. Mellen , and J. Seidensticker . 1993. “The Influence of Food Presentation on the Behavior of Small Cats in Confined Environments.” Zoo Biology 12, no. 2: 203–216.

[zoo21857-bib-0023] Stander, P. E. 1992. “Foraging Dynamics of Lions in a Semi‐Arid Environment.” Canadian Journal of Zoology, 70, no. 1: 8–21.

[zoo21857-bib-0024] Stanton, L. A. , M. S. Sullivan , and J. M. Fazio . 2015. “A Standardized Ethogram for the Felidae: A Tool for Behavioral Researchers.” Applied Animal Behaviour Science 173: 3–16.

[zoo21857-bib-0025] Stein, A. B. , S. L. Bourquin , and J. W. McNutt . 2015. “Avoiding Intraguild Competition: Leopard Feeding Ecology and Prey Caching in Northern Botswana.” African Journal of Wildlife Research 45, no. 2: 247–257.

[zoo21857-bib-0026] Suárez, P. , P. Recuerda , and L. Arias‐de‐Reyna . 2017. “Behaviour and Welfare: The Visitor Effect in Captive Felids.” Animal welfare 26, no. 1: 25–34.

[zoo21857-bib-0027] Sunquist, M. E. 2010. “Tigers: Ecology and Behavior.” In Tigers of the World, edited by R. L. Tilson and P. J. Nyhus , 19–33. San Diego: Elsevier.

[zoo21857-bib-0028] Sunquist, M. , and F. Sunquist . 2002. Wild Cats of the World. London, United Kingdom: The University of Chicago Press.

[zoo21857-bib-0029] Sunquist, M. E. 1981. “The Social Organization of Tigers (Panteratigris) in Royal Chitawan National Park.” Nepal Smith Contrib Zoology, 336: 1–98.

[zoo21857-bib-0030] Ulyan, M. J. , A. E. Burrows , C. A. Buzzell , M. A. Raghanti , J. L. Marcinkiewicz , and K. A. Phillips . 2006. “The Effects of Predictable and Unpredictable Feeding Schedules on the Behavior and Physiology of Captive Brown Capuchins (Cebus apella).” Applied Animal Behaviour Science 101, no. 1–2: 154–160.

[zoo21857-bib-0031] Waitt, C. , and H. M. Buchanan‐Smith . 2001. “What Time Is Feeding?” Applied Animal Behaviour Science 75, no. 1: 75–85.

[zoo21857-bib-0032] Watters, J. V. 2014. “Searching for Behavioral Indicators of Welfare in Zoos: Uncovering Anticipatory Behavior.” Zoo Biology 33, no. 4: 251–256. 25042907 10.1002/zoo.21144

