# Supplementary material for: Effects of Random Feeding Schedule on Pacing in Asiatic Lions (Panthera leo persicus)
Source: Zoo Biol. 2024 Sep 30;43(6):590–4. doi: 10.1002/zoo.21857 (PMC11624625; doi:10.1002/zoo.21857)
Supplement: Supplementary file 1 — Supplementary information. [file ZOO-43-590-s001.docx]

Supplementary Material

**Table S1:** Lion ethogram, based on standardized felid ethogram (Stanton et al., 2015)

| **Category** | **Behavior** | **Behavior description** |
| --- | --- | --- |
| **Active** | Standing | Cat is in an upright position and immobile, with all four paws on the ground and legs extended, supporting the body |
|  | Walking | Forward locomotion at a slow gait, which is slower than trotting |
|  | Trotting | Forward locomotion at a swift gait performed with alternating steps. Movement is faster than walking but slower than running |
|  | Running | Forward locomotion in a rapid gait, which is faster than walking or trotting |
|  | Social play | Cat interacts with another cat in a “non-serious” manner (i.e., where there is no intention to harm) |
|  | Individual play | Cat interacts with an object in a “non-serious” manner (i.e., where there is no intention to harm) or cat rolls onto its back, with its belly exposed and all paws in the air, within a playful context. |
| **Inactive** | Lying | Cat’s body is on the ground in a horizontal position, including on its side, back, belly, or curled in a circular formation. |
|  | Huddling | Cat is at rest, lying or sitting with body in contact with another cat. |
|  | Sitting | Cat is in an upright position, with the hind legs flexed and resting on the ground, while front legs are extended and straight |
| **Feeding** | Eating | Cat ingests food (or other edible substances) by means of chewing with the teeth and swallowing. |
|  | Drinking | Cat ingests water (or other liquids) by lapping up with the tongue. |
| **Pacing** | Pacing | Repetitive locomotion in a fixed pattern, such as back and forth along the same route. Can include walking, trotting and running. Movement seems to have no apparent goal or function. Must be performed at least three times in succession before qualifying as stereotypic. |
| **Other** | Out of sight | Cat is not visible to observer |
|  | Other | Cat shows behavior that does not fit into any of the abovementioned categories |

**Figure S1.** Mean lion activity budgets under the fixed and random feeding schedule. Categories represent mean proportion of scans observed in a category over all scan samples. Total daily observation time: six hours, 360 scan samples.

**Figure S2.** Mean lion activity budgets per individual lion under the fixed and random feeding schedule. Categories represent mean proportion of scans observed in a category over all scan samples. Total daily observation time: six hours, 360 scan samples.
